# Millimeter-Wave Body-Centric Radar Sensing for Continuous Monitoring of Human Gait Dynamics

**DOI:** 10.3390/s26061844

**Published:** 2026-03-15

**Authors:** Yoginath Ganditi, Mani S. Chilakala, Zahra Najafi, Mohammed E. Eltayeb, Warren D. Smith

**Affiliations:** Department of Electrical and Electronic Engineering, California State University, Sacramento, CA 95819, USA; yoginathganditi@csus.edu (Y.G.); manisaichilakala@csus.edu (M.S.C.); zahra.najafi@csus.edu (Z.N.); mohammed.eltayeb@csus.edu (M.E.E.)

**Keywords:** gait analysis, millimeter-wave radar, FMCW radar, micro-Doppler, spatiotemporal gait parameters, step detection, corridor-based monitoring, fixed (tripod-mounted) radar, shoe-mounted radar, digital health

## Abstract

Gait is a sensitive marker of mobility decline and fall risk, motivating unobtrusive sensing methods that can extract spatiotemporal parameters outside specialized gait laboratories. This paper presents a physics-based comparison of two millimeter-wave frequency-modulated continuous-wave (FMCW) radar deployment paradigms using a low-cost, system-on-chip (SoC) 60 GHz Infineon BGT60TR13C radar sensor: (i) a fixed (tripod-mounted) corridor observer and (ii) a shoe-mounted body-centric configuration attached to the medial side of the left shoe. Four healthy adult author-participants performed repeated 30 s corridor trials under five gait styles (regular, slow, fast, simulated festination, and simulated freezing-of-gait), including brief pauses during turns; an empty-corridor recording was acquired to characterize static clutter. Step events were detected using peak-picking on foot-related velocity envelopes with adaptive thresholds, and step count, cadence, step time, and step-time variability were derived. Performance of the fixed and shoe-mounted configurations was quantitatively compared to video ground truth using mean absolute percentage error (MAPE) for step count estimation. Across all gait styles, the shoe-mounted FMCW radar consistently reduced step-count error relative to the fixed corridor-mounted configuration, with the largest gains under irregular patterns (e.g., festination: 37.1% fixed vs. 9.6% shoe-mounted). These findings highlight the advantages of body-centric millimeter-wave radar sensing and support low-cost SoC radar as a pathway toward wearable, privacy-preserving gait monitoring in real-world environments.

## 1. Introduction

Continuous gait monitoring provides objective indicators of mobility decline, fall risk, and disease progression, yet most clinical gait assessments remain infrequent and are typically performed in controlled environments using structured protocols [1,2]. Gold-standard approaches such as instrumented gait laboratories and optical motion-capture systems offer high accuracy but are costly, infrastructure intensive, and difficult to deploy at scale. Camera-based alternatives reduce cost but raise privacy concerns and remain sensitive to lighting, occlusions, and installation constraints. Wearable inertial sensors and pressure insoles enable ambulatory monitoring, but they require direct body contact, frequent charging or calibration, and sustained user compliance, which can limit long-term adoption [3,4]. These limitations motivate sensing modalities that are unobtrusive, privacy-preserving, and suitable for continuous use in everyday environments.

Beyond dedicated radar systems, recent device-free radio-frequency (RF) approaches have leveraged existing ambient infrastructure (e.g., commodity Wi-Fi and smart speakers) for indoor tracking and gait-based recognition or identification. Representative examples include STAGR, WSTrack+, and XModal-ID, which demonstrate strong performance but typically require multiple fixed transceivers and environment-specific calibration, and, in some cross-modal identification settings, may be evaluated against candidate video footage [5,6,7]. In contrast, mmWave radar provides a self-contained sensing modality that is privacy-preserving (no identifiable imagery), robust to lighting and many occlusions, and can complement or reduce dependence on on-body inertial sensing for long-term monitoring [3,4]. Doppler radar has emerged as a compelling alternative because it is contactless, robust to lighting conditions, and capable of capturing motion dynamics through Doppler and micro-Doppler signatures [1,8]. Recent advances in millimeter-wave (mmWave) radar further enhance these capabilities by enabling fine range discrimination through wide bandwidths, spatial selectivity through compact antenna arrays, and rich micro-Doppler structure for characterizing periodic limb motion [9,10]. As a result, mmWave sensing has gained increasing attention in healthcare and ambient assisted living, supporting unobtrusive monitoring of human activity in natural environments [9,11]. Prior work has demonstrated the feasibility of extracting clinically relevant gait parameters such as cadence and stride timing from radar micro-Doppler signatures, including in in-home and real-world deployments [1,2,8,12].

The clinical relevance of continuous gait monitoring is well established, as gait abnormalities are hallmark manifestations of neurological and musculoskeletal disorders and can serve as early indicators of disease progression or treatment response [3,13]. Parkinson’s disease (PD), for example, is commonly associated with short shuffling steps, reduced stride length, festination, and freezing-of-gait (FOG) [3,14]. Post-stroke gait often exhibits hemiplegic asymmetry with compensatory patterns such as spasticity or dragging of the paretic limb [13,15]. Other conditions, including multiple sclerosis (MS) and cerebral palsy (CP), can produce spastic, ataxic, or crouched gait patterns that disrupt speed, symmetry, and stride timing [13]. Despite the diversity of these impairments, many clinically meaningful signatures are reflected in spatiotemporal measures such as step timing and its variability, underscoring the need for accurate and continuous monitoring outside the clinic [3,16].

In this work, we investigate a shoe-mounted 60 GHz system-on-chip (SoC) frequency-modulated continuous-wave (FMCW) radar for continuous gait monitoring. Operation at 60 GHz (λ≈5 mm) enhances sensitivity to small, rapid foot motions and enables rich micro-Doppler signatures arising from the foot–ground interaction region. At the same time, SoC integration consolidates the RF front end and baseband processing into a compact, low-power platform suitable for wearable deployment. Importantly, body-centric placement removes the reliance on a fixed line-of-sight geometry and reduces susceptibility to bystanders, corridor clutter, and environmental multipath compared with environment-mounted radar systems [8,11].

While fixed indoor radars have been widely studied for unobtrusive gait analysis [1,2,8], and early feasibility of wearable radar sensing has been demonstrated at lower carrier frequencies (e.g., K-band) [17], direct comparisons between fixed and shoe-mounted *millimeter-wave* radar using the *same* low-cost SoC sensor and a unified processing pipeline remain limited. This gap is particularly evident under gait styles that emulate clinically relevant abnormalities, such as festination and freezing-of-gait, where step timing becomes compressed or intermittent and robust step extraction is challenging. To address these gaps, this paper makes the following contributions:

- We present and evaluate a shoe-mounted, body-centric 60 GHz FMCW SoC radar for gait monitoring and directly compare it with a fixed corridor-mounted deployment using identical sensor hardware and a unified signal-processing pipeline.
- We propose a unified, deterministic signal-processing pipeline for FMCW radar gait analysis, enabling consistent step-event extraction from both fixed and shoe-mounted deployments.
- We extract and analyze step count and spatiotemporal gait parameters, including cadence, step time, and step-time variability, across multiple gait styles, and quantitatively validate step counting against video ground truth using mean absolute percentage error (MAPE).

By enabling a portable, objective, and privacy-preserving sensing modality, the proposed shoe-mounted mmWave radar approach offers a promising pathway toward continuous, real-world gait assessment, with potential applications in early detection of gait deterioration, rehabilitation monitoring, and long-term mobility tracking outside the clinic.

## 2. Materials and Methods

This section describes the radar hardware, data-collection protocol, and deterministic signal-processing pipeline used to extract step events and spatiotemporal gait parameters from raw 60 GHz FMCW radar measurements. The central methodological contribution is a controlled comparison between *fixed corridor* and *shoe-mounted body-centric* sensing using the *same* low-cost SoC radar and a *single unified processing pipeline*. By holding the sensor and algorithms constant, observed performance differences can be attributed primarily to sensing geometry.

### 2.1. Radar Hardware and FMCW Configuration

All measurements were acquired using a 60 GHz Infineon BGT60TR13C FMCW radar (BGT60TR13C shield), Infineon Technologies AG, Neubiberg, Bavaria, Germany. Two deployment geometries were evaluated: (i) a tripod-mounted radar observing a corridor walking path and (ii) a shoe-mounted radar attached to the medial side of the left shoe. Device configuration files (JSON) for both setups are released with the dataset, and radar operation follows the manufacturer documentation [18,19].

Table 1 summarizes the FMCW waveform parameters. The fixed corridor configuration uses a bandwidth of B=2 GHz, yielding a range resolution of $\Delta R\approx \frac{c}{2B}\approx 7.5$ cm, appropriate for corridor-scale observation. The shoe-mounted configuration uses a wider bandwidth (B≈5 GHz) to achieve finer near-range resolution (ΔR≈3.0 cm), enabling clearer separation of inter-foot reflections. The sampling rate and chirp timing determine the approximate unambiguous range and maximum unambiguous radial velocity, which motivate deployment-specific range gating during processing.

### 2.2. Participants and Data-Collection Protocol

Four healthy adult author–participants are denoted P1–P4, where P*i* refers to the *i*th author-participant in the dataset. Participants completed corridor walking trials under five gait styles selected to include both steady and clinically relevant irregular patterns: regular, slow, higher cadence (fast), simulated festination (short rapid steps), and simulated freezing-of-gait (brief episodic pauses or shuffling). Each trial lasted 30 s, during which participants walked back and forth along a 5 m corridor segment and turned naturally at the endpoints, introducing brief pauses and turn-induced transients typical of hallway assessments. An empty-corridor recording (no subject present) was also collected to support background and clutter suppression.

**Figure 1:**
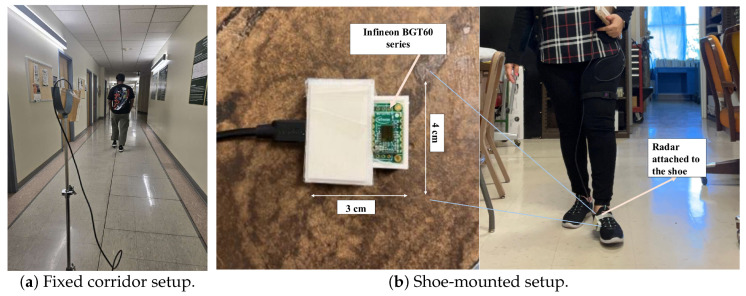
Radar deployments. (**a**) Tripod-mounted fixed radar in the corridor. (**b**) Shoe-mounted radar attached on the medial side of the left foot.

Figure 1 shows the fixed corridor and shoe-mounted radar deployments. Both deployments use the same 60 GHz SoC FMCW radar and the same gait-parameter extraction pipeline; therefore, observed performance differences are attributed primarily to sensing geometry and the resulting micro-Doppler contrast in the velocity–time (VT) representation, rather than to differences in hardware or signal processing.

**Fixed corridor deployment.** The radar is mounted on a tripod facing along the corridor walking direction. This environment-referenced geometry captures whole-body motion together with corridor-dependent clutter and multipath. During processing, a corridor-specific range-of-interest (ROI) is applied to emphasize the walking lane and suppress strong static returns from walls and background structures.

**Shoe-mounted deployment.** The radar is attached to the medial side of the left shoe and oriented toward the contralateral foot during swing. This body-centric geometry emphasizes near-field inter-foot reflections and produces alternating signed Doppler components as the contralateral foot approaches and recedes relative to the sensor, typically enhancing step-related contrast in the VT representation.

### 2.3. Unified Signal-Processing Pipeline

**Figure 2:**
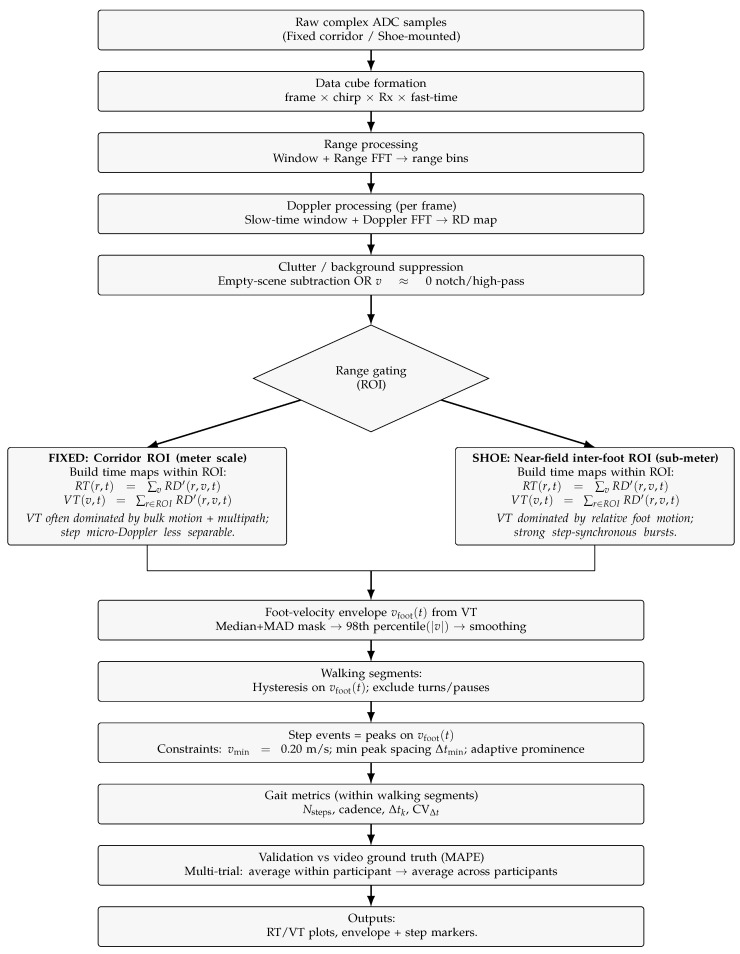
Unified processing pipeline for both deployments. RD maps are formed from raw ADC data followed by clutter suppression and deployment-specific range gating. RT/VT maps are computed within the ROI and step events are detected from peaks in a VT-derived foot-velocity envelope.

A single deterministic signal-processing pipeline is used for both deployments, as summarized in Figure 2. All processing was implemented in MATLAB (R2025b, The MathWorks, Inc., Natick, MA, USA). The pipeline begins with raw complex ADC samples arranged as x[t,k,m]∈C, where $t\in \{1,\ldots ,N_{f}\}$ indexes frames, $k\in \{1,\ldots ,N_{c}\}$ indexes chirps, and $m\in \{1,\ldots ,N_{s}\}$ indexes fast-time samples. A windowed FFT over *m*, followed by a windowed FFT over *k*, yields a range–Doppler (RD) map RD(r,v,t) with range bins $r\in \{1,\ldots ,N_{r}\}$ and Doppler (velocity) bins $v\in \{1,\ldots ,N_{v}\}$. Static clutter is suppressed either by subtraction of an empty-scene estimate ${\hat{RD}}_{0}\left(r,v\right)$ or by removing bins near zero radial velocity (v≈0), producing the clutter-suppressed map $RD^{\prime }\left(r,v,t\right)$. The RD maps are subsequently range-gated to a deployment-specific region of interest (ROI) $R\subseteq \{1,\ldots ,N_{r}\}$ which includes a corridor ROI for the fixed radar and a near-field inter-foot ROI for the shoe-mounted radar. Within R, range–time (RT) and velocity–time representations are obtained by marginalization as follows

(1) $$\begin{array}{cc} RT\left(r,t\right)=\sum_{v=1}^{N_{v}}RD^{\prime }\left(r,v,t\right),\;r\in R;\;\mathrm{and}\;VT\left(v,t\right) & =\sum_{r\in R}RD^{\prime }\left(r,v,t\right),v\in \left\{1,\ldots ,N_{v}\right\}. \end{array}$$

### 2.4. Step Timing and Walking-Segment Selection

A foot-velocity envelope $v_{\mathrm{foot}}\left(t\right)$ is extracted from VT(v,t) using a per-frame robust mask M(t) (median+MAD, where MAD denotes the median absolute deviation) and an upper-tail percentile *p* (e.g., p=98), followed by light smoothing to obtain

(2) $$v_{\mathrm{foot}}\left(t\right)={\mathrm{percentile}}_{p}\left(\;|v|\;:\;v\in M(t)\right).$$

Walking intervals $\left\{W_{j}\right\}$ are detected via hysteresis on $v_{\mathrm{foot}}\left(t\right)$ (minimum durations; turns/pauses excluded). Step events are peaks in $v_{\mathrm{foot}}\left(t\right)$ with constraints $v_{\min}$, $\Delta t_{\min}$, and adaptive prominence. Metrics are computed only from step times within $\cup_{j}W_{j}$ (Segment-Constrained Evaluation). We deliberately apply the same deterministic envelope-and-peak strategy to both deployments in order to isolate the impact of sensing geometry; more sophisticated sequence-alignment or multi-scale temporal post-processing techniques, such as dynamic time warping and multi-scale analysis of gait trajectories, could be layered on top of the VT or step-interval series in future work [20,21].

### 2.5. Gait Metrics and Validation

Let $T_{\mathrm{walk}}=\sum_{j}\left|W_{j}\right|$. From step times $\left\{t_{k}\right\}\subset \cup_{j}W_{j}$, define $\Delta t_{k}=t_{k+1}-t_{k}$ and compute

(3) $$\begin{array}{cc} N_{\mathrm{steps}} & =\sum_{j}\left|\{t_{k}\in W_{j}\}\right|, \end{array}$$

(4) $$\begin{array}{cc} \mathrm{cadence} & =60\;\frac{N_{\mathrm{steps}}}{T_{\mathrm{walk}}}, \end{array}$$

(5) $$\begin{array}{cc} {\mathrm{CV}}_{\Delta t} & =100\;\frac{\sigma (\Delta t_{k})}{\mu (\Delta t_{k})}. \end{array}$$

Video provides $N_{\mathrm{steps}}^{GT}$ and step-count error as

(6) $$\mathrm{MAPE}=100\cdot \frac{|N_{\mathrm{steps}}-N_{\mathrm{steps}}^{GT}|}{N_{\mathrm{steps}}^{GT}}.$$

### 2.6. Computational Complexity and Runtime Characteristics

The dominant computational cost of the proposed pipeline arises from the fast-time and slow-time fast Fourier transforms (FFTs) used to form range–Doppler maps for each frame. For a data cube with $N_{f}$ frames, $N_{c}$ chirps per frame, and $N_{s}$ fast-time samples per chirp, the combined range and Doppler processing has complexity to the order of $O(N_{f}N_{c}N_{s}\log N_{s}+N_{f}N_{s}N_{c}\log N_{c})$. Subsequent operations—clutter suppression, deployment-specific range gating, RT/VT marginalization, formation of the foot-velocity envelope, hysteresis-based walking-segment detection, and peak-based step-event extraction—are linear in the number of time samples and therefore do not change the overall asymptotic complexity relative to the FFT blocks. In practice, similar two-stage FFT pipelines already execute in real time on low-power processors integrated with mmWave radar system-on-chip platforms, and the additional envelope and peak-detection logic incurs only a modest overhead compared with the front-end signal processing [9,10]. Consequently, the overall algorithm is compatible with real-time or near real-time execution for the waveform parameters and frame rates summarized in Table 1.

## 3. Experimental Results and Analysis

### 3.1. Representative RT/VT Maps and Mechanistic Interpretation for Regular Walking

We begin with representative range–time and velocity–time maps to illustrate how measurement geometry affects step visibility. In our pipeline, step events are extracted from peaks in a foot-related velocity envelope derived from VT (Section 2); therefore, the separability and periodicity of step-related velocity bursts in VT provide a mechanistic explanation for step-count performance.

**Figure 3:**
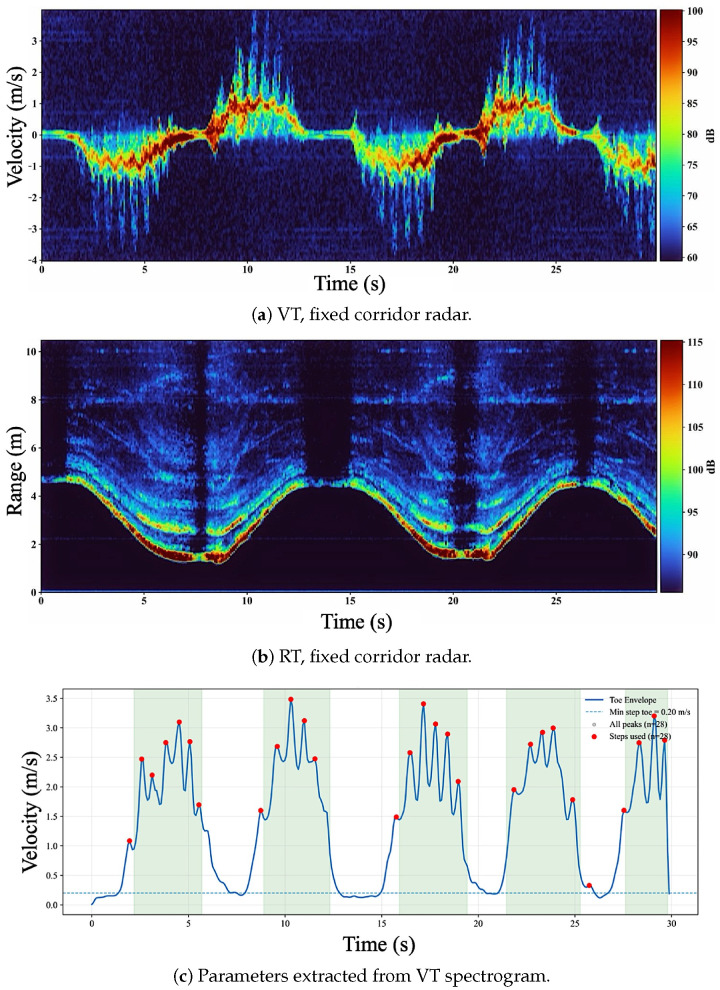
Fixed corridor radar measurements for participant P1 during regular walking: (**a**) VT spectrogram showing bulk Doppler with limb micro-Doppler and sign reversal during the turn as the subject walks toward and then away from the radar; (**b**) RT intensity map showing periodic range migration during out-and-back corridor motion; (**c**) step extraction from the VT-derived foot-velocity envelope using $v_{\min}=0.20$ m/s, with walking segments (shaded) and detected steps (red).

Figure 3 shows fixed-corridor radar measurements for participant P1 during regular walking. The VT map in Figure 3a is dominated by a smooth bulk-motion Doppler ridge whose sign reverses with walking direction. Weaker limb micro-Doppler components are present but are partially masked by whole-body motion and residual corridor clutter, reducing the contrast of step-synchronous bursts. Consistent with this bidirectional motion, the RT map in Figure 3b exhibits repeated “U-shaped” range trajectories corresponding to successive out-and-back corridor traversals, with deceleration near each turnaround.

Figure 3c illustrates step-event extraction from the VT-derived foot-velocity envelope. A minimum peak threshold ($v_{\min}=0.20$ m/s) suppresses low-velocity fluctuations, and physiological timing constraints reject spurious detections. Shaded regions indicate automatically detected walking segments used for computing cadence and step-time variability, excluding brief pauses at corridor turns. Compared with shoe-mounted sensing, the fixed-corridor envelope exhibits reduced peak separability due to dominant bulk motion, increasing ambiguity in step detection and contributing to higher step-count error in later results.

**Figure 4:**
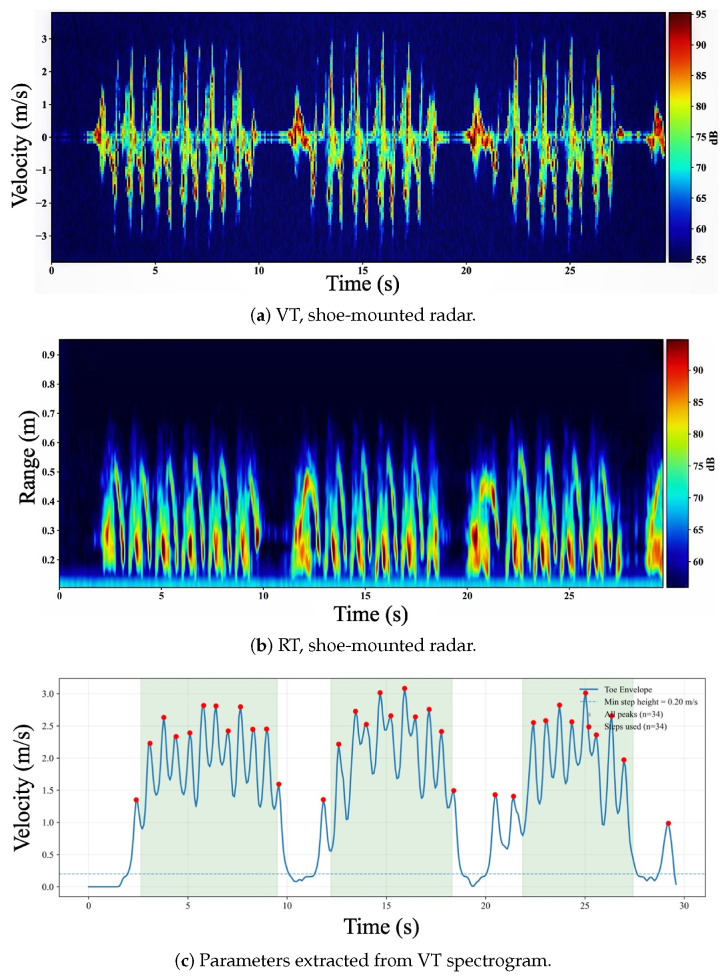
Shoe-mounted radar measurements for participant P1 during regular walking. (**a**) Velocity–Time (VT) spectrogram showing high-contrast periodic foot micro-Doppler. (**b**) Range–Time (RT) intensity map with energy concentrated at short ranges (≈0.1–0.3 m) due to body-centric inter-foot motion. (**c**) Step-event extraction from the VT-derived foot-velocity envelope using a minimum velocity threshold ($v_{\min}=0.20$ m/s); retained steps (red) and detected walking segments (shaded).

Figure 4 shows representative shoe-mounted measurements for participant P1 during regular walking. The VT map in Figure 4a exhibits high-contrast, regularly spaced Doppler bursts with components on both sides of zero velocity. These alternating signed components arise from the contralateral foot approaching and receding relative to the shoe-mounted radar within each gait cycle. As the measurement is body-referenced and dominated by near-field relative motion, the VT representation is largely decoupled from corridor multipath and bulk body translation, yielding a cleaner micro-Doppler structure than in the fixed corridor configuration.

The RT map in Figure 4b concentrates energy at short (sub-meter) ranges, consistent with the near-field inter-foot geometry. During swing, the contralateral foot repeatedly enters the radar field of view and produces strong returns at characteristic short ranges, while returns weaken during stance as the relative geometry becomes more static.

Figure 4c illustrates step-event extraction from the shoe-mounted VT signal. The foot-related velocity envelope $v_{\mathrm{foot}}\left(t\right)$ (solid) is obtained via robust masking and high-percentile selection, producing periodic peaks that align with repeatable high-velocity phases of the gait cycle (typically contralateral swing relative to the instrumented shoe). A minimum peak-height threshold ($v_{\min}=0.20$ m/s, dashed) suppresses noise-driven fluctuations and residual low-velocity motion, and physiological timing constraints reject spurious detections. The retained step events are shown in red, and shaded regions denote automatically detected walking segments used for computing cadence and step-time variability; brief pauses at corridor turns are excluded. In this example, the detected peaks are well separated and stable over time, resulting in 28 retained steps over the 30 s trial and illustrating the high step-to-background contrast achieved by body-centric sensing.

Across both deployments (Figure 3 and Figure 4), gait parameters are computed from step-event times $\left\{t_{k}\right\}$ obtained primarily from VT, while RT serves as a supporting diagnostic to verify target presence within the intended range region, guide configuration-specific range gating, and assist walking-segment detection for excluding turns and pauses. Mechanistically, the shoe-mounted geometry improves step detectability by measuring near-field *relative* motion between the instrumented shoe and the contralateral foot, producing step-synchronous Doppler bursts that are less influenced by corridor multipath or whole-body translation. In contrast, the fixed corridor view integrates returns from multiple body segments and residual environmental clutter, and VT can be dominated by bulk translation, which can mask foot micro-Doppler and reduce peak separability, particularly under higher cadence and short, rapid steps.

### 3.2. Higher-Cadence Walking (P3): Step Bursts Remain Separable in Shoe-Mounted VT

**Figure 5:**
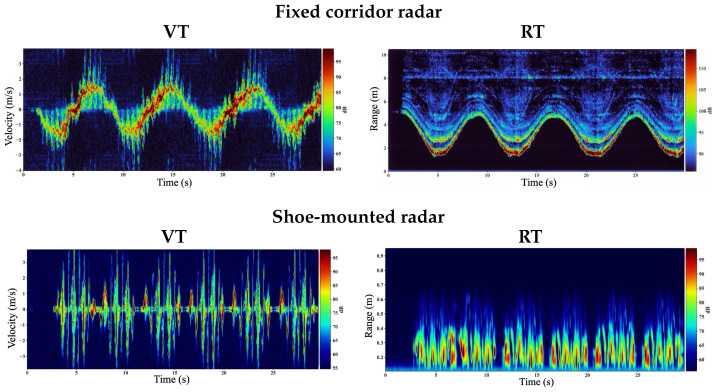
Representative higher-cadence walking trial for participant P3 comparing fixed corridor radar (**top**) and shoe-mounted radar (**bottom**). In the fixed VT spectrogram, bulk whole-body Doppler dominates and step-related micro-Doppler is less separable at higher cadence. In the shoe-mounted VT, near-field relative foot motion produces high-contrast, step-synchronous Doppler bursts that yield a cleaner envelope for peak-based step detection.

Figure 5 compares the fixed corridor and shoe-mounted RT/VT maps for a representative higher cadence trial from participant P3. A first difference is visible directly in the fixed RT map where the subject completes each corridor lap in under ∼10 s, compared with ∼14 s for regular walking trials, confirming an increased translation speed and shorter turnaround period. This faster range migration increases the magnitude of the dominant bulk-motion component in VT (whole-body radial velocity) and reduces the time available for step-related limb micro-Doppler to remain isolated.

In the fixed corridor VT, the strongest energy concentrates along a smooth, continuous ridge associated with torso/hip translation, while step-related micro-Doppler components appear as weaker modulations that are partially masked by the increased bulk Doppler at higher speed. Consequently, the VT-derived foot-velocity envelope used by the step detector (Section 2) exhibits lower step-to-background contrast, which increases peak ambiguity (missed peaks and/or spurious detections) as cadence increases.

In contrast, the shoe-mounted VT remains dominated by near-field *relative* motion between the instrumented shoe and the contralateral foot. The result is a sequence of high-contrast, regularly spaced Doppler bursts that remain separable even when the overall walking pace increases. As these bursts produce a cleaner, more periodic foot-velocity envelope, peak-based step timing remains robust under higher cadence in the shoe-mounted configuration. The corresponding shoe RT map also remains concentrated at short ranges (inter-foot distances), indicating that the sensed dynamics are governed by gait-cycle kinematics rather than corridor-scale range migration. Overall, Figure 5 provides a mechanistic explanation for the observed accuracy advantage of shoe-mounted sensing under faster, higher-cadence walking where body-centric geometry preserves step-synchronous micro-Doppler structure needed for reliable event detection.

### 3.3. Festination (P2): Fixed Corridor VT Degrades Under Short, Rapid Steps

Festination is characterized by a rapid cadence with shortened step length and reduced swing amplitude. From a radar perspective, these changes compress the temporal spacing between step-related micro-Doppler bursts and can reduce the peak limb radial velocity. As a result, festination is a challenging condition for step extraction, particularly for a fixed corridor observer where the received signal is a mixture of whole-body translation, swinging-limb micro-Doppler, and residual corridor clutter.

**Figure 6:**
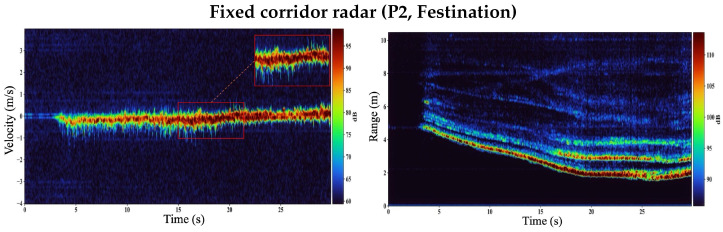
Fixed corridor radar during simulated festination for participant P2. (**Left**): VT map, where step-related micro-Doppler is weak and less separable due to dominance of whole-body radial motion. (**Right**): RT map showing overall range migration during walking; while the target trajectory is visible, individual steps are difficult to isolate from RT alone under this geometry.

Figure 6 shows VT and RT heatmaps for participant P2 during a representative festination trial using the fixed radar. The RT map exhibits the expected slow range migration associated with the participant walking toward and away from the radar, including gradual changes in range around turning intervals. However, the corresponding VT map is dominated by a broad, high-energy bulk-motion component (torso/upper-body radial velocity) and residual clutter contributions within the corridor ROI. Step-related micro-Doppler components appear as weaker, intermittent energy spread around the bulk ridge, but under festination they become less separable in time and less distinct in magnitude. This reduced step-to-background contrast leads to ambiguity when forming the VT-derived foot-velocity envelope $v_{\mathrm{foot}}\left(t\right)$ and increases the likelihood of missed or spurious peaks during peak-based step detection.

**Figure 7:**
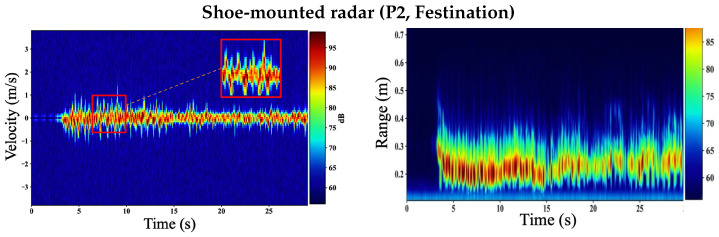
Shoe-mounted radar (P2, Festination). (**Left**): VT map exhibiting clearer, more periodic swing-related Doppler bursts driven by near-field inter-foot relative motion, improving step-event separability for envelope-and-peak detection. (**Right**): RT map with energy concentrated at short ranges consistent with contralateral foot returns.

Figure 7 shows the corresponding shoe-mounted VT/RT heatmaps for the same gait style. In the shoe-mounted geometry, the radar measures near-field *relative* motion between the instrumented shoe and the contralateral foot. Consequently, the VT map typically contains clearer, regularly repeating swing-related Doppler bursts that remain visible even when steps are short and rapid.

Because the gait pipeline extracts step events from peaks in $v_{\mathrm{foot}}\left(t\right)$ derived from VT, this increased micro-Doppler contrast in the shoe-mounted case produces more reliable peak timing and reduces step-count error under festination.

**Figure 8:**
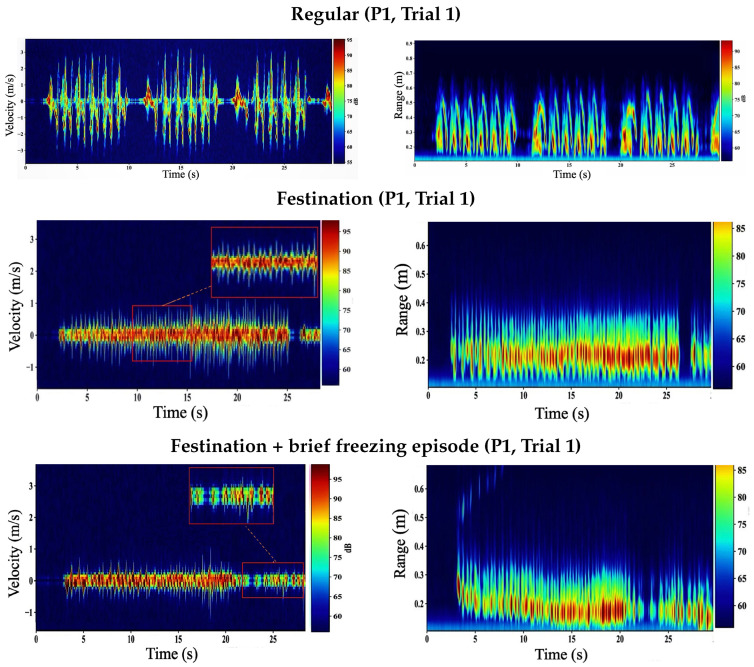
Shoe-mounted VT (**left**) and RT (**right**) for participant P1 (Trial 1) comparing regular walking, simulated festination, and a festination trial containing a brief freezing episode (festination → freeze → festination).

Figure 8 compares shoe-mounted velocity–time (VT) and range–time (RT) maps for participant P1 (Trial 1) under three walking patterns: regular walking, simulated festination, and a festination trial that includes a brief freezing episode (festination–freeze–festination). In the shoe-mounted geometry, the radar primarily observes *near-field relative motion* between the instrumented shoe and the contralateral foot. Consequently, step-related micro-Doppler signatures remain high-contrast and time-localized across all three conditions, which directly benefits envelope-and-peak step extraction from VT.

*Turn-around effect in VT:* Across shoe-mounted trials, turn-around/repositioning intervals between straight-walking passes can introduce sustained higher-|v| VT energy; these segments are removed by walking-segment selection prior to toe-envelope formation and peak-based step extraction.

During regular walking, VT exhibits stable, evenly spaced Doppler bursts corresponding to the swing phase of the contralateral foot. These bursts create a smooth and repeatable foot-related velocity envelope $v_{\mathrm{foot}}\left(t\right)$ with well-separated local maxima, enabling reliable estimation of step-event times $\left\{t_{k}\right\}$ and derived temporal gait parameters such as step count, cadence, and the surrogate step-time sequence $\Delta t_{k}=t_{k+1}-t_{k}$. The corresponding RT map is concentrated at short ranges (sub-meter), consistent with inter-foot distances observed by the shoe-mounted sensor; repeated near-range return structures align with the swing-cycle geometry.

Under festination, step timing compresses and the inter-burst spacing in VT decreases, producing a higher “burst density” over time. Despite shorter and faster steps, the shoe-mounted VT retains clear periodic structure because it measures relative foot motion with reduced sensitivity to corridor clutter and whole-body translation. As a result, peaks in $v_{\mathrm{foot}}\left(t\right)$ remain separable, preserving step-event timing even when cadence increases and swing amplitude may decrease.

During the festination trial containing a brief freezing episode, the VT map shows predominantly festination-like stepping for the first ∼22 s, characterized by dense, closely spaced swing-related Doppler bursts (compressed inter-burst spacing). A short interval then appears in which these swing bursts largely vanish and VT energy collapses toward low velocities for approximately 2–3 s, consistent with a brief intentional freezing episode. Immediately afterward, the burst pattern reappears and returns to a festination-like cadence as stepping resumes. In the proposed pipeline, the hysteresis-based walking-segment selector marks the near-stationary interval as non-walking, preventing spurious peak detections; step-event timing and summary metrics are computed from the steady stepping intervals before and after the freeze. When motion restarts, the re-emerging Doppler bursts again yield separable maxima in the VT-derived envelope, allowing peak-based step extraction to recover cleanly after the brief pause.

### 3.4. Step-Count Error (MAPE) Against Video Ground Truth

**Figure 9:**
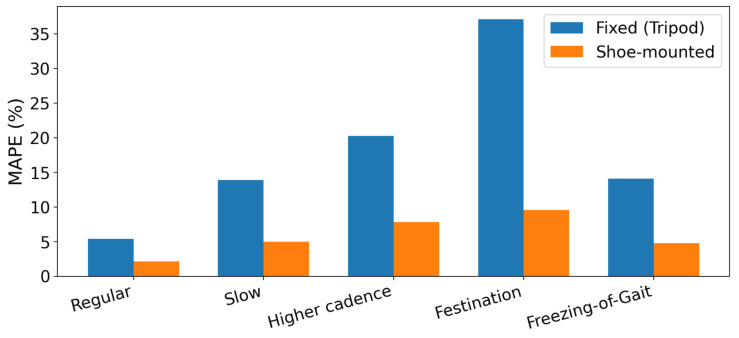
Step-count error by gait style for fixed (tripod-mounted) versus shoe-mounted 60 GHz FMCW radar. Bars show mean absolute percentage error (MAPE, %) between detected and video ground-truth step counts, aggregated across participants (P1–P4).

Figure 9 summarizes step-count accuracy across five gait styles using mean absolute percentage error (MAPE) relative to video ground truth. The shoe-mounted configuration achieves lower error than the fixed corridor radar in every condition: regular (2.1% vs. 5.3%), slow (5.0% vs. 13.9%), higher-cadence (7.8% vs. 20.3%), festination (9.6% vs. 37.1%), and freezing-of-gait (4.8% vs. 14.1%). The largest separation occurs during festination, where short, rapid steps compress swing signatures and reduce peak separability in the corridor-facing VT, while the shoe-mounted VT retains strong, periodic swing-related bursts. Overall, the consistent reduction in MAPE supports the hypothesis that body-centric near-field sensing improves step-event reliability by mitigating corridor multipath/clutter and reducing sensitivity to dominant bulk-motion Doppler components that can mask limb micro-Doppler in the fixed deployment.

### 3.5. Per-Participant Metrics (Step Count, Cadence, and Variability)

Table 2 reports per-participant results for detected step count ($N_{\mathrm{steps}}$), ground-truth step count ($N_{\mathrm{steps}}^{\mathrm{GT}}$), cadence, and step-time variability (${\mathrm{CV}}_{\Delta t}$) across gait styles. For P1 and P2, festination and freezing-of-gait were repeated for three trials; all other conditions used one trial. As fixed and shoe recordings are acquired as separate trials, the video ground-truth step counts can differ between deployments; therefore, $N_{\mathrm{steps}}^{\mathrm{GT}}$ is reported as Fixed/Shoe (F/S) in the same format as the detected metrics.

## 4. Discussion and Limitations

The quantitative step-count results summarized in Figure 9, together with the mechanistic RT/VT examples in Section 3, indicate that shoe-mounted body-centric sensing yields a robust micro-Doppler representation of step events across regular, higher-cadence, and irregular gait styles, including simulated festination and freezing-of-gait. Across all four participants and five gait conditions, the shoe-mounted configuration achieved equal or lower step-count mean absolute percentage error than the fixed corridor-mounted radar, with the largest relative gains occurring under irregular, pathological-like patterns where bulk translation and corridor clutter most strongly mask limb micro-Doppler in the fixed view. Compared with established laboratory-grade gait measurement systems such as marker-based optical motion capture or instrumented walkways, the proposed mmWave approach offers substantially less kinematic detail but requires only a compact single-chip sensor, minimal infrastructure, and no cameras or body markers. Optical motion-capture and pressure-plate systems remain the gold standard for ground-truth gait kinematics in clinical and biomechanics research; future work will incorporate concurrent recordings with such systems to enable direct comparison of stride-length and joint-level parameters beyond the step-timing metrics considered here.

Relative to IMU- and MEMS-based wearable systems for gait anomaly and freezing-of-gait detection [3,22], shoe-mounted mmWave radar provides a contactless, privacy-preserving alternative that reduces dependence on user adherence and fixed line-of-sight constraints while still leveraging step timing and its variability as clinically meaningful markers. Device-free RF systems such as STAGR, WSTrack+, and XModal-ID further demonstrate that commodity Wi-Fi and smart-speaker infrastructure can be exploited for indoor tracking and gait-based identification across rooms and even through walls, but they typically rely on multiple access points or speakers and introduce distinct privacy considerations due to their coupling with video footage [5,6,7]. Despite these promising findings, the present study has several important limitations.

First, the sample size is small (N=4), and the participants were the authors, which restricts statistical power and limits the generalizability of the reported error statistics. Second, festination and freezing-of-gait were simulated by healthy author–participants rather than observed in patients with Parkinson’s disease or other neurological disorders, so the resulting patterns may not fully capture the complexity or variability of true pathological gait. Third, all recordings were conducted in a single straight corridor with controlled conditions, and video-based step annotations served as the only ground truth; gold-standard systems such as VICON motion capture or instrumented walkways were not available for this dataset. Finally, the current pipeline focuses on a deterministic envelope-and-peak detector and does not yet integrate more advanced sequence-alignment, multi-scale, or learning-based post-processors, which could further improve robustness under highly irregular or noisy conditions and could be used to cross-validate the deterministic approach adopted here [20,21]. Accordingly, the present work should be interpreted as a controlled, physics-based comparison of sensing geometries using identical hardware and algorithms, rather than as a definitive clinical validation study. Future research will extend this body-centric mmWave framework to larger and more diverse cohorts, including participants with documented gait impairments, and will benchmark radar-derived gait parameters directly against optical motion capture, instrumented walkways, and state-of-the-art inertial and device-free RF systems.

## 5. Conclusions and Future Work

This paper compared a fixed corridor-mounted and a medial-side shoe-mounted 60 GHz FMCW radar for gait monitoring using a low-cost SoC sensor, the same deterministic processing pipeline, and the same protocol across four participants (P1–P4) and five gait styles. Across all conditions, the shoe-mounted configuration reduced step-count error (MAPE) relative to the fixed corridor deployment (Figure 9), with the largest improvement during festination where short, rapid steps compress swing signatures in the corridor view but remain discernible in the shoe-mounted VT due to near-field relative-motion sensing.

Mechanistically, shoe mounting converts an environment-referenced measurement that mixes bulk translation, limb micro-Doppler, and corridor clutter into a body-referenced measurement dominated by inter-foot relative motion, yielding higher contrast and more step-synchronous Doppler structure for envelope-and-peak step detection. While these results indicate a clear geometry advantage under the tested conditions, the study is limited by a small cohort and a controlled corridor setup; broader validation across environments, shoe placements, and clinical populations is needed. Future work will focus on an embedded, low-power shoe-worn implementation for real-time operation, larger multi-session datasets, and benchmarking against established gait measurement systems.

## Figures and Tables

**Table 1:** FMCW waveform parameters for fixed and shoe-mounted recordings (from device configuration JSON).

Parameter	Fixed (Tripod)	Shoe-Mounted
Start frequency (GHz)	61.000	58.250
End frequency (GHz)	63.000	63.248
Bandwidth (GHz)	2.000	4.998
Samples per chirp	280	64
Sampling rate (MS/s)	1.006	2.000
Chirps per frame	128	128
Chirp repetition time $T_{r}$ (ms)	0.300	0.247
Frame repetition time (ms)	77.269	77.269
Range resolution $\Delta R\approx \frac{c}{2B}$ (m)	0.075	0.030
Approx. unambiguous range (m)	∼10.49	∼0.96
Approx. max. unambiguous speed (m/s)	∼4.03	∼4.99

**Table 2:** Fixed (tripod-mounted) vs. shoe-mounted 60 GHz FMCW radar gait metrics across multiple gait styles for four participants (P1–P4), reported as Fixed/Shoe (F/S). Metrics include detected and ground-truth step counts, cadence, and step-time variability ${\mathrm{CV}}_{\Delta t}$. Festination and Freezing-of-Gait were repeated for three trials for P1 and P2; all other conditions used a single trial.

Participant	Gait Style	Trials	$N_{\mathrm{steps}}$	$N_{\mathrm{steps}}^{\mathrm{GT}}$	Cadence	${\mathrm{CV}}_{\Delta t}$ (%)
P1	Regular	1/1	28/34	30/34	62.8/76.1	85.5/55.1
P1	Slow	1/1	28/28	25/28	63/63.8	50.9/37.3
P1	Fast	1/1	30/28	40/32	68/63.6	91.8/99.4
P1	Festination	1/3	44/64	72/69	91.5/92.5	42.1/38.7
P1	Festination	2/3	43/65	68/71	91.6/96.7	36.8/34.7
P1	Festination	3/3	42/69	73/70	91.2/100.7	32.5/52.1
P1	Freezing-of-Gait	1/3	48/68	53/63	109.5/100.1	57.6/37.3
P1	Freezing-of-Gait	2/3	46/70	54/67	96.4/100.1	55.9/41.9
P1	Freezing-of-Gait	3/3	49/57	52/61	100.5/82.4	41.7/39.3
P2	Regular	1/1	39/36	41/35	83.1/85	72.9/82.0
P2	Slow	1/1	27/30	29/29	56.6/74.4	45.0/43.6
P2	Fast	1/1	38/39	49/41	78.4/88.6	78.3/97.7
P2	Festination	1/3	44/72	69/76	102.9/107.7	35.8/48.8
P2	Festination	2/3	46/64	72/69	99.4/95.6	45.8/52.3
P2	Festination	3/3	49/66	75/70	103.6/94.9	51.1/55.8
P2	Freezing-of-Gait	1/3	37/61	51/64	78.8/90.4	87.0/52.0
P2	Freezing-of-Gait	2/3	43/65	41/68	94.2/93.5	55.3/64.2
P2	Freezing-of-Gait	3/3	44/80	62/77	95.3/115.7	51.4/45.7
P3	Regular	1/1	36/37	35/35	83.4/78.2	38.3/38.9
P3	Slow	1/1	33/36	26/39	67.5/81.5	44.2/25.4
P3	Fast	1/1	43/37	52/40	94.8/88.9	50.1/56.0
P3	Festination	1/1	37/48	77/55	78.6/114	48.9/42.8
P3	Freezing-of-Gait	1/1	49/47	54/50	100.8/108.6	41.4/45.4
P4	Regular	1/1	39/40	42/40	82.4/85.6	56.6/25.4
P4	Slow	1/1	28/31	31/34	63.9/64.4	17.5/25.4
P4	Fast	1/1	36/43	43/46	82.4/91.2	48.2/43.0
P4	Festination	1/1	55/50	70/58	113.2/109.7	51.5/36.9
P4	Freezing-of-Gait	1/1	42/43	36/42	87.6/92.5	26.7/18.8

## Data Availability

The data presented in this study were generated by the authors for system development and validation purposes and are not publicly available. Additional information may be provided by the corresponding author upon reasonable request.

## Abbreviations

FMCW	Frequency-Modulated Continuous Wave
mmWave	Millimeter-Wave
RD	Range–Doppler
RT	Range–Time
VT	Velocity–Time
μD	Micro-Doppler
GT	Ground Truth
MAPE	Mean Absolute Percentage Error
MAD	Median Absolute Deviation
FOG	Freezing of Gait
IMU	Inertial Measurement Unit
MCU	Microcontroller Unit
